# Does antenatal magnesium sulphate improve hearing function in premature newborns?

**DOI:** 10.4274/jtgga.galenos.2019.2019.0070

**Published:** 2020-09-03

**Authors:** Işıl Kasapoğlu, Bilge Çetinkaya Demir, Mehmet Aral Atalay, Adnan Orhan, Hilal Özkan, Salih Cağrı Çakır, Rabia Tütüncü Toker, Fikret Kasapoğlu, Kemal Özerkan

**Affiliations:** 1 Department of Obstetrics and Gynecology, Bursa Uludağ University Faculty of Medicine, Bursa, Turkey; 2 Department of Pediatrics, Bursa Uludağ University Faculty of Medicine, Bursa, Turkey; 3 Department of Otorhinolaryngology, Bursa Uludağ University Faculty of Medicine, Bursa, Turkey

**Keywords:** Magnesium sulphate, prematurity, hearing screening, hearing loss, ototoxicity, neuroprotection

## Abstract

**Objective:**

To evaluate whether antenatal magnesium sulphate (MgSO4) exposure has a neuroprotective effect against hearing impairment in premature newborns.

**Material and Methods:**

Retrospective cohort study was performed with prematurely (<37 weeks) delivered newborns at a tertiary university hospital. Newborns of 92 women who received MgSO4 infusions (study group) for various indications were compared to newborns of 147 women who did not receive MgSO4 infusions (control group). All eligible premature newborn underwent hearing screening by auditory brainstem response (ABR) testing before being discharged from the hospital.

**Results:**

The fail rate for ABR hearing screening was 3.3% (n=3) in the study group and 10.9% (n=16) in the control group (p=0.034). The rate of concurrent use of betamethasone was higher in the study group (72.8%; n=67) compared to control group (29.2%; n=43) (p<0.001). Other neonatal parameters, such as the number of neonates who were small for gestational age and the rate of microcephaly were similar between the groups (p=0.54, p=0.48, respectively). After adjusting for co-variates including the use of betamethasone and gestational age at delivery, no statistically significant association between antenatal administration of MgSO4 and ABR fail rates were found (p=0.07).

**Conclusion:**

These results do not suggest a significant benefit in terms of hearing impairment in premature newborns when antenatal MgSO4 infusion was given.

## Introduction

Currently, magnesium sulphate (MgSO4) is widely used in obstetric care for various indications to improve obstetric outcomes. These include a reduction in the risk of eclampsia and as a tocolytic agent, although its efficacy as a tocolytic agent is still controversial. A recent suggestion has been that MgSO4 may be neuroprotective for the immature fetal central nervous system and may reduce the incidence of major neurological morbidity, particularly cerebral palsy (CP) in premature newborns (1,2).

The earliest data on the use of MgSO4 for neuroprotection was published in the 1980s and 1990s (3,4). Data were collected from infants who were exposed to MgSO4 in utero. In those cases, perinatal morbidity including intracranial events and CP were observed to be less severe (3,4). According to those preliminary findings, magnesium has been investigated widely for its neuroprotective effects and furthermore, guidelines have been created, especially for antenatal administration of MgSO4 (AAM) in premature deliveries (1). MgSO4 infusions have been proposed to protect the fetal central nervous system in preterm infants and also to reduce the rate of preterm births in patients presenting with threatened preterm labor.

Recent advances in intensive care techniques have been associated with improved survival rates in premature infants (5). However, the rate of preterm birth complications has not yet been reduced (6). Currently, prematurity is the leading cause of the central nervous system morbidities (7). Major neurological disorders accompanying prematurity include CP, mental retardation, sensorineural hearing loss (SNHL), and blindness (8,9). In spite of decreased rates of prematurity-related mortality, the incidence of SNHL has remained high, varying from 0.2% to 6.4% (10). Hearing loss, which also impairs speech and language development, is a major disability closely associated with social and physical development of the newborn, and has a significant impact on the quality of life. Congenital hearing loss is a universal health problem and is one of the health measurements which is used to determine health-related quality of life (11). Early recognition and adequate treatment regimens can mitigate adverse outcomes. Hence, newborn hearing screening programmes have been widely introduced and hearing screening is recommended in all infants during the first month after delivery (12).

There is a lack of evidence-based data on the efficacy of AAM in preventing hearing loss in preterm infants. Most previous studies assessing magnesium usage for neuroprotection have evaluated CP as the primary outcome (2), but there are insufficient data on cochlear function. There are some studies which suggest a beneficial effect of magnesium administration on noise induced hearing loss in adults (13). However the mechanism of noise induced hearing loss and the mechanism of congenital hearing loss are different. Considering the widespread use of AAM in obstetric care, the aim of the present study was to assess the potential neuroprotective effect of antenatal MgSO4 on auditory nerve development and sensorineural hearing in premature newborns.

## Material and Methods

This study was performed in a tertiary university hospital in Turkey, from January 2015 to January 2017. The study protocol was approved by the Local Research Ethics Committee (Uludağ University Faculty of Medicine Clinical Research Ethics Committee) at the beginning of the study (approval number: 2017-14/12). Each participant was informed about the study and provided their written consent.

All preterm infants (<37^th^ gestational weeks) born within this two year were included in the study. Medical records of study subjects were retrospectively reviewed. Neonates born to mothers who were administered antenatal MgSO4 for at least eight hours before the delivery for various indications as shown below, were enrolled in the study group. Neonates whose mothers did not receive MgSO4 constituted the control group. Among them, newborns who had a history of intrauterine infections, lethal congenital abnormalities, craniofacial anomalies, and who died after birth were excluded from the study.

Antenatal MgSO4 (magnesium sulfate 15%; Biofarma) was administered by continuous intravenous infusion at a rate of 2 grams per hour. Infusion duration was at least 8 hours, following a loading dose of 4.5 grams, as recommended. Indications for MgSO4 included neuroprotection, tocolysis or prophylaxis for eclampsia. Antenatal administration of corticosteroids consisted of two courses of 12 mg betamethasone, when indicated.

The primary outcome measurement was the failure rate on auditory brain stem (ABR) hearing screening in newborns. ABR allows neurophysiologic assessment of brainstem maturation and auditory pathway in newborns. The effect of antenatal MgSO4 infusion was the main variable.

As all of the newborns were premature and had risk factors for hearing loss, all eligible newborns underwent ABR hearing screening during the first month after delivery, before being discharged from the neonatal intensive care unit (NICU). The results of ABR tests were evaluated and compared between the study and control groups. ABR tests were performed using Madsen Accuscreen (Otometrics, Denmark). The ABR consisted of up to 30 dB click stimulus using disposable, hydrogel electrodes and the standard for the device was EN-60645-7, type 2.

Based on the results of screening ABR test, newborns were considered to have “passed the test”, if the stimulus to produce a response did not exceed 30 decibels (dB-NHL) in both ears; if not, they were considered to have “failed the test”, according to the suggested algorithm of hearing screening of newborns.

Maternal characteristics which could affect neonatal outcomes, such as maternal age, parity, use of antenatal corticosteroids, presence of preeclampsia, premature rupture of membranes (PROM), delivery route and neonatal characteristics including gestational age at birth, birth weight, Apgar scores, and umbilical cord arterial pH values were also retrieved. In addition, other neonatal parameters evaluated within the first 28 days, such as microcephaly and the incidence of postnatal morbidities including requirement for mechanical ventilation, neonatal sepsis and exposure to ototoxic agents, meningitis, intraventricular hemorrhage, retinopathy of prematurity, respiratory distress syndrome (RDS), phototherapy requirement, and bronchopulmonary dysplasia were analyzed as composite outcomes. The rate of pathological electroencephalography (EEG), visual evoked potentials and early Denver Developmental Screening test results were also analyzed.

### Statistical analysis

The SPSS version 21.0 (IBM Corp., Armonk, NY., USA) was used to analyze study data. The distribution pattern of the data was examined for normality with the Shapiro-Wilk test. Variables were reported as mean ± standard deviation or median (minimum-maximum) values. According to the normality test result, an independent sample t-test or Mann-Whitney U test were used for intergroup comparisons. The chi-square test or Fisher’s exact test was used for the comparisons of categorical variables. Binary logistic regression analysis was performed in order to determine independent risk factors that may have affected failure rates in the ABR screening test. The level of significance was set at α=0.05.

## Results

All infants born before 37^th^ week were evaluated. During the study period 285 eligible women were administered MgSO4 and/or gave birth before 37^th^ week of gestation. Newborns who died after birth (n=37, 12.9%) and whose hearing screening results were not available (n=9, 3.1%) were excluded from the study. A total of 239 newborns were included in further statistical analysis. Mothers of 92 out of 239 newborns (38.5%) received MgSO4 whereas mothers of 147 (61.5%) newborns did not.

The mean maternal age at delivery was similar between the study group and the control group (30.5±5.9 versus 30.7±5.7 years respectively, p=0.83). Cesarean delivery rate was significantly higher in the study group (80.4%) compared to the control group (68.8%) (p=0.048) (Table 1).

Demographic and clinical characteristics of the cohort groups are shown in Table 1. The median (range) gestational age at delivery was significantly lower in the study group compared to the control group at 32 (26-36^+3^) and 35 (26^+5^-36^+5^) weeks, respectively (p<0.001) and the mean birth weight was lower in the study group compared to that in the control group (1733±586 g and 2336±668 g, respectively; p<0.001). The incidence of PROM was similar in the two groups (2.4% versus 5.1%, p=0.29). The prevelence of preeclampsia was significantly higher in the study group compared to the control group (41.3% versus 3.4%, p<0.001). Apgar scores at one and five minutes were lower in the newborns in study group than those in control group (p=0.017 and p=0.03, respectively). There was no significant difference between two groups in the umbilical cord pH measurements (p=0.23) (Table 1).

Nineteen newborns failed the ABR screening test, in total. Failure rates significantly differed between the two groups with 3.3% (n=3) in the study group and 10.9% (n=16) in the control group, respectively, failing the test (p=0.034) (Table 2). Seven out of 19 (36.8%) infants who failed in the ABR test had unilateral hearing loss and 12 (63.2%) had bilateral loss.

The rate of antenatal administration of betamethasone (AAB) was significantly higher in the study group (72.8%) compared to the control group (29.2%) (p<0.001). No statistically significant association was found between AAB and the result of ABR hearing screening (p=0.31).

The rates of small for gestation age neonates and the incidence of microcephaly did not significantly differ between the two groups (p=0.54, p=0.48, respectively). Composite neonatal outcome, with the exception of RDS, was similar in the groups (Table 3). Additionally, the rate of pathological EEGs, visual evoked potentials and the results of early Denver Developmental Screening test and being treated in NICU did not significantly differ between the two groups (Table 3).

Adjustments were analyzed using a logistic regression model for AAM, AAB, and gestational age (day) as major variables significantly differ between groups and that could be associated with ABR hearing screening outcomes. In this final model, none of the variables was found to be an independent variable for ABR results (Table 4).

## Discussion

The use of MgSO4 is mainly recommended for the prophylaxis of eclampsia and recently, for the protection of fetal central nervous system in preterm labor before 32 weeks (1). The aim of the present study was to evaluate whether AAM exposure had a neuroprotective effect on hearing in premature newborns. Our results showed that the failure rate in the ABR screening test was lower in the group of newborns exposed to antenatal MgSO4 than in the group of neonates who were not exposed to antenatal MgSO4 (p=0.034). Nevertheless, after adjustment for covariates, including betamethasone and gestational age at delivery (day), there was no statistically significant association between AAM and the failure rate in ABR screening (p=0.07).

Magnesium is an essential element for many physiological processes in the body (14). One of these functions is the maintenance of cell membrane polarization by regulating calcium channels (15). In this context, magnesium contributes to cochlear physiology and has a role in hearing process. Despite its known neuroprotective effects and protective effects against CP, data on the effect of magnesium on sensorineural hearing is limited. In a study conducted with guinea pigs, a negative correlation was observed between cochlear magnesium levels and hearing loss. It was demonstrated that magnesium content of the inner ear has a regulatory function and hinders reactive oxygen species formation after noise exposure (16,17). Furthermore, there is evidence of cellular damage due to oxidative stress associated with magnesium deficiency (18). In addition, in adults, the protective effects of oral magnesium supplementation have been demonstrated in hearing loss due to acoustic trauma (13). Most of these studies were conducted with animals or adults and particularly evaluated noise-induced cochlear damage. This evidence prompted the present study to evaluate the possible protective effects of magnesium on sensorineural hearing in premature newborns. Premature newborns were specifically evaluated, because of the higher incidences of SNHL in this population (19), which is related to delayed neurodevelopment (12,19).

In line with previous studies, we found a high percentage of hearing impairment as assessed by the ABR screening test (7.9%) in the whole cohort of premature newborns. Although various mechanisms could be suggested for the development of hearing loss in premature newborns, prematurity alone is one of the established major risk factors (19) as fetal audiological development mainly occurs between 20 and 33 weeks of gestation. Therefore, the risk for labyrinth pathologies of the inner ear is also increased in premature newborns (20). This was the reasoning behind including premature newborns in this study.

Other etiologic factors include hypoxic damage, mechanical ventilation, hemorrhage, and increased levels of bilirubin and administration of ototoxic drugs such as aminoglycoside antibiotics. In a previous study, which investigated risk factors for hearing loss in preterm newborns, it was suggested that the etiology of hearing loss was multifactorial rather than a single factor that has a prominent effect on ototoxicity (21). Thus clinical factors were evaluated in two groups separately in this study. Intergroup comparisons indicated that newborns in the study group who received antenatal MgSO4 had significantly lower birth weights and lower one minute and five minute Apgar scores. This could be related with the earlier gestational week at birth. However, they performed better in the hearing screening test than newborns who did not received antenatal MgSO4.

A recent Cochrane review, which evaluated the potential neuroprotective effect of antenatal magnesium in preterm newborns, included a limited number of studies (2). Furthermore, most of these studies evaluated mortality, gross motor disability or CP as primary outcomes. Mortality rates did not differ with magnesium pretreatment with a relative risk of 1.04 (95% confidence invertal: 0.92-1.17) (2). In our study population, we also found a very high mortality rate (12.9%), supporting that prematurity was a risk factor. However newborns died after birth, were not included in the further statistical analysis.

To date, there are not enough data to interpret the protective effect of magnesium administration on sensorineural hearing. Only one study, included in the above review, evaluated neurological impairment in 1047 participants (22). In that study, hearing loss was not separately evaluated but was instead accepted as a component of moderate neurological disability. The authors did not report significant difference between groups of patients who received magnesium and who did not, with respect to sensorineural disability (22).

One previous retrospective study evaluated the outcomes of hearing screening for any possible associations with antenatal maternal medications. The maternal medication list did not include magnesium. Corticosteroid use was reported to be associated with a reduced risk for hearing loss (23). It should be noted that in most of the studies which evaluated the neuroprotective effects of magnesium, participants also received corticosteroids, as was the case in the present study. In another recent study, which only evaluated the effects of antenatal steroid administration on hearing function in newborns, no associations were found between corticosteroids and hearing screening results (24). In a further study repeated antenatal administration of steroids rather than a single dose did not add benefit based on ABR evaluations in newborns (25). Moreover in another study, it was indicated that steroid dosage made no difference in hearing function of newborns born after the 34th gestational week (26). In our study population, we also used betamethasone to improve neonatal outcomes. In line with those studies, we did not find any direct association between antenatal betamethasone administration and neonatal ABR hearing screening test results (p=0.31) in univariate, as well as further multivariate analyses.

Preliminary results of this study of AAM indicated improved hearing screening test results in premature newborns in our cohort. However, further multivariate analysis did not support the initial results. Therefore, there is no robust data indicating a clear benefit from AAM in terms of hearing impairment in premature newborns. This study has significant implications for hearing loss in premature newborns as a high-risk population for SNHL and also suggests that AAM is a possible adjuvant therapy that may reduce the incidence of SNHL in this group.

In our study population we detected a higher incidence of impaired hearing based on screening outcomes in neonates born after 34 weeks of gestation compared to preterm infants who had received antenatal magnesium. A possible explanation for this result might be the potential protective effect of magnesium on cochlear functions or on the fetal brain, which might still remain important in neonates born after 34 weeks of gestation. However possible confounders cannot be excluded. Any discrepancy in such association needs to be elucidated with further studies.

Although this study included all premature newborns (<37 weeks) over a period of two years, this is a relatively small study population and the retrospective nature of the study was a further limitation of this study. Additionally, for the newborns who were treated in NICUs before undergoing the ABR test, we could not exclude possible further external confounding factors.

## Conclusion

Our results do not suggest a clear and definite benefit from antenatal MgSO4 infusion in respect of hearing impairment in premature newborns. For the usage of MgSO4 as a neuroprotective medication against hearing impairment in premature newborns, further large scale and carefully designed studies are warranted to reach a definite conclusion.

## Figures and Tables

**Table 1:**
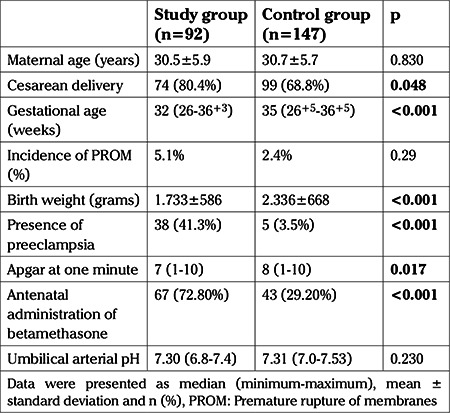
Demographic and clinical characteristics of the cohort groups

**Table 2:**
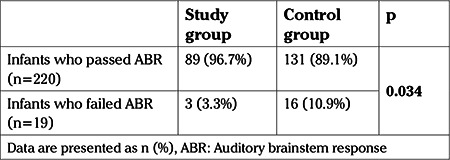
Auditory brainstem response hearing screening outcomes with respect to antenatal administration of magnesium sulphate

**Table 3:**
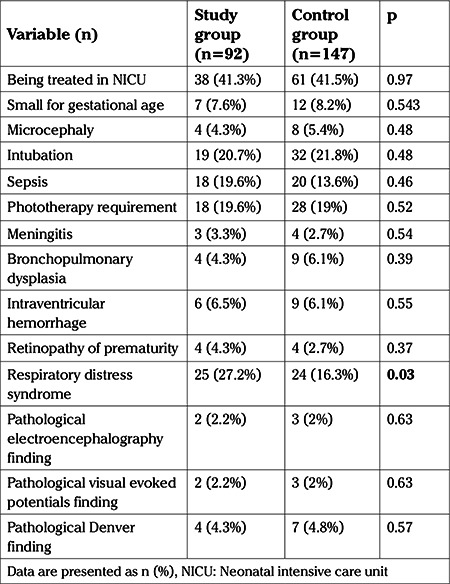
Neonatal parameters and postnatal morbidities in the study and control groups

**Table 4:**
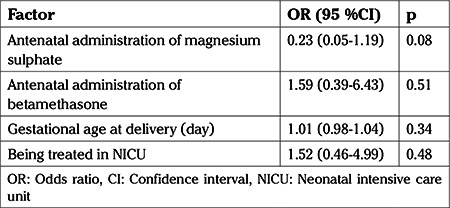
Logistic regression model of variables associated with the auditory brainstem response hearing screening test results

## References

[1] No authors listed (2013). American College of Obstetricians and Gynecologists Committee on Obstetric Practice Society for Maternal-Fetal Medicine. Committee Opinion No. 573: Magnesium sulfate use in obstetrics. Obstet Gynecol.

[2] Doyle LW, Crowther CA, Middleton P, Marret S, Rouse D (2009). Magnesium sulfate for women at risk of preterm birth for neuroprotection of the fetus. Cochrane Database Syst Rev.

[3] van de Bor M, Verloove-Vanhorick SP, Brand R, Keirse MJ, Ruys JH (1987). Incidence and prediction of periventricular-intraventricular hemorrhage in very preterm infants. J Perinat Med.

[4] Kuban KC, Leviton A, Pagano M, Fenton T, Strassfeld R, Wolff M (1992). Maternal toxemia is associated with reduced incidence of germinal matrix hemorrhage in premature babies. J Child Neurol.

[5] Ward RM, Beachy JC (2003). Neonatal complications following preterm birth. BJOG.

[6] Nelson KB (2003). Can we prevent cerebral palsy?. N Engl J Med.

[7] Wen SW, Smith G, Yang Q, Walker M (2004). Epidemiology of preterm birth and neonatal outcome. Semin Fetal Neonatal Med.

[8] Fazzi E, Orcesi S, Caffi L, Ometto A, Rondini G, Telesca C, et al (1993). Neurodevelopmental outcome at 5-7 years in preterm infants whith periventrikular leucomalatia. Neuropediatrics.

[9] Knoches AM, Doyle LW (1993). Long-term outcome of infants born preterm. Baillienes Clin Obstet Gynaecol.

[10] Martínez-Cruz CF, García Alonso-Themann P, Poblano A, Ochoa-López JM (2012). Hearing loss, auditory neuropathy, and neurological co-morbidity in children with birthweight <750 g. Arch Med Res.

[11] Horsman J, Furlong W, Feeny W, Torrence G (2003). The Health Utilities Index (HUI): concepts, measurement properties and applications. Health Qual Life Outcomes.

[12] No authors listed (1993). Early identification of hearing impairment in infants and young children. NlH Consens Statement.

[13] Attias J, Weisz G, Almog S, Shahar A, Wiener M, Joachims Z, et al (1994). Oral magnesium intake reduces permanent hearing loss induced by noise exposure. Am J Otolaryngol.

[14] Mildvan AS (1987). Role of magnesium and other divalent cations in ATP-utilizing enzymes. Magnesium.

[15] Horie M, Irisawa H, Noma A (1987). Voltage-dependent magnesium block of adenosine-triphosphate-sensitive potassium channel in guinea-pig ventricular cells. J Physiol.

[16] Xiong M, Wang J, Yang C, Lai H (2013). The cochlea magnesium content is negatively correlated with hearing loss induced by impulse noise. Am J Otolaryngol.

[17] Sendowski I (2006). Magnesium therapy in acoustic trauma. Magnes Res.

[18] Freedman AM, Mak IT, Stafford RE, Dickens BF, Cassidy MM, Muesing RA, et al (1992). Erythrocytes from magnesium-deficient hamsters display an enhanced susceptibility to oxidative stress. Am J Physiol.

[19] Robertson C, Sauve RS, Christianson HE (1994). Province based study of neurologic disability among survivors weighing 500 through to 1249 g at birth. Pediatrics.

[20] Höing R (1991). Inner ear changes in fetal asphyxia. Laryngorhinotologie.

[21] Marlow ES, Hunt LP, Marlow N (2000). Sensorineural hearing loss and prematurity. Arch Dis Child Fetal Neonatal Ed.

[22] Crowther CA, Hiller JE, Doyle LW, Haslam RR (2003). Australasian Collaborative Trial of Magnesium Sulphate (ACTOMg SO4) Collaborative Group. Effect of Magnesium sulfate given for neuroprotection before pretem birth. A randomized controlled trial. JAMA.

[23] J Leung JC, Cifra CL, Agthe AG, Sun CC, Viscardi RM (2016). Antenatal factors modulate hearing screen failure risk in preterm infants. Arch Dis Child Fetal Neonatal Ed.

[24] Waters TP, Silva N, Denney JM, Sciscione AC, Paul DA (2008). Neonatal hearing assessment in very low birth weight infants exposed to antenatal steroids. J Perinatol.

[25] Church MW, Wapner RJ, Mele LM, Johnson F, Dudley DJ, Spong CY, et al (2010). Repeated courses of antenatal corticosteroids: Are there effects on the infant’s auditory brainstem responses?. Neurotoxicol Teratol.

[26] Amin SB, Guillet R (2007). Auditory neural maturation after exposure to multiple courses of antenatal betamethasone in premature infants as evaluated by auditory brainstem response. Pediatrics.

